# CHD1, a multifaceted epigenetic remodeler in prostate cancer

**DOI:** 10.3389/fonc.2023.1123362

**Published:** 2023-01-26

**Authors:** Haoyan Li, Loraine Gigi, Di Zhao

**Affiliations:** ^1^ Department of Experimental Radiation Oncology, The University of Texas MD Anderson Cancer Center, Houston, TX, United States; ^2^ Texas A&M School of Public Health, Texas A&M University, College Station, TX, United States

**Keywords:** CHD1, prostate cancer, epigenetic remodeler, dysregulation, therapeutic strategy

## Abstract

Chromatin remodeling proteins contribute to DNA replication, transcription, repair, and recombination. The chromodomain helicase DNA-binding (CHD) family of remodelers plays crucial roles in embryonic development, hematopoiesis, and neurogenesis. As the founding member, CHD1 is capable of assembling nucleosomes, remodeling chromatin structure, and regulating gene transcription. Dysregulation of CHD1 at genetic, epigenetic, and post-translational levels is common in malignancies and other human diseases. Through interacting with different genetic alterations, CHD1 possesses the capabilities to exert oncogenic or tumor-suppressive functions in context-dependent manners. In this Review, we summarize the biochemical properties and dysregulation of CHD1 in cancer cells, and then discuss CHD1’s roles in different contexts of prostate cancer, with an emphasis on its crosstalk with diverse signaling pathways. Furthermore, we highlight the potential therapeutic strategies for cancers with dysregulated CHD1. At last, we discuss current research gaps in understanding CHD1’s biological functions and molecular basis during disease progression, as well as the modeling systems for biology study and therapeutic development.

## Introduction

Chromatin remodeling is a major regulator of gene expression. Chromatin remodelers utilize ATP hydrolysis to slide the nucleosomes onto and off the DNA, thereby regulating the accessibility of genes to a range of nuclear factors, including transcriptional factors (1). Chromatin remodeling proteins contribute to DNA recombination, transcription, repair, and replication (2). Based on the similarities and differences in catalytic ATPases and associated subunits, chromatin remodelers can be classified into four subfamilies: Imitation Swtich (ISWI), Chromodomain Helicase DNA-binding (CHD), Switch/sucrose Non-fermentable (SWI/SNF) and INO80 (3). The CHD family comprises nine members and plays crucial roles in embryonic development, hematopoiesis, and neurogenesis (2, 4, 5). Notably, nearly all CHD members are dysregulated and mutated in human malignancies. Increasing evidence points to the roles of CHD members during cancer development and progression. Through promoting the transcription of oncogenes or tumor suppressor genes, some CHD enzymes possess the capability to exert both oncogenic and tumor-suppressive functions in context-dependent manners.

CHD1 is the founding member of the CHD family and is conserved across all eukaryotes (6). CHD1 is capable of assembling nucleosomes, remodeling chromatin structure, modulating histone turnover, and regulating gene transcription (7–9). In embryonic stem cells (ESCs), CHD1 is a key regulator of open/loose chromatin, correlates with a permissive transcriptional state, and directly contributes to developmental pluripotency characteristics (10–13). The induction of CHD1 expression is also essential in the programming of the pluripotent stem cells (5). In the past decade, large-scale cancer genome studies showed recurrent deletions of the *CHD1* gene in ~8% of prostate cancer (14–20). In prostate tumors, loss of CHD1 causes DNA repair defects, androgen receptor (AR) redistribution and dysfunction, chromatin instability, and transcriptional plasticity (21–24). However, in PTEN-deficient prostate tumors, the CHD1 protein is stabilized and contributes to cancer progression, tumor microenvironment remodeling, and drug resistance (25–27).

In this Review, we focus on the chromatin remodeler CHD1 that plays multifaceted roles in prostate cancer. We summarize CHD1’s biochemical properties and dysregulation in cancer cells, as well as discuss its biological functions in different contexts of prostate cancer, emphasizing its crosstalk with diverse signaling pathways. In addition, we highlight the differential therapeutic strategies for cancers harboring CHD1 defects or overexpression.

## Biochemical and structural properties of CHD1

**Figure 1:**
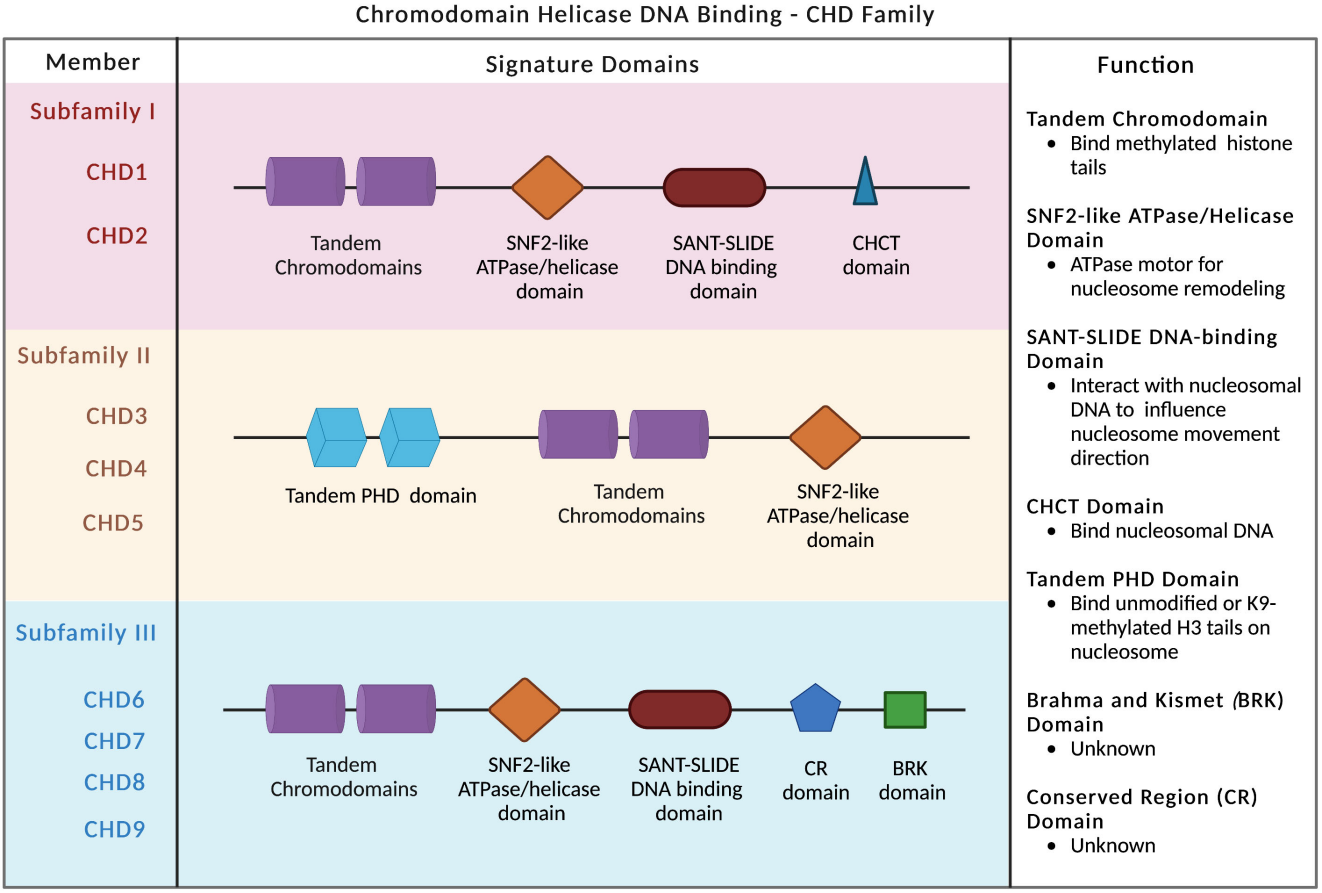
CHD family and signature domains. Three subfamilies of CHD chromatin remodelers are presented with signature domains. The major functions of each domain are listed.

Compared to other chromatin remodelers, the CHD family is distinguished by two signature motifs: tandem chromodomains located in the N-terminal region and the SNF2-like ATP-dependent helicase domain centered in the middle of the protein (28) (**Figure 1**). The chromodomains bind to methylation marks on histones, while the SNF2-like ATPase domain confers enzymatic activity and regulates nucleosome remodeling and chromatin conformational change (2, 29). Based on the constituent domains, CHD proteins are classified into three subfamilies: subfamily I (CHD1/2), subfamily II (CHD3–5), and subfamily III (CHD6–9) (**Figure 1**). In addition to chromodomains and ATPase domain, CHD1 and CHD2 proteins also contain SANT-SLIDE DNA-binding domains located in the C-terminal region (**Figure 1**), and preferentially bind to AT-rich DNA motifs (28, 30, 31). Although CHD1 and CHD2 are highly homologous to one another, they are significantly divergent in the 3′ regions and may possess distinct functions. In contrast, subfamily II proteins (CHD3–5) are distinguished by N-terminal tandem PHD (plant homeodomain) Zn finger-like domains (**Figure 1**). They are core components of the nucleosome remodeling and histone deacetylase complex (NuRD) (32). The third subfamily (CHD6–9) is evolutionarily conserved and contains additional featured domains, such as the Brahma and Kismet domain (BRK), the conserved region (CR) domains, and the SANT-SLIDE-like domain (**Figure 1**).

The chromatin association specificity of CHD proteins is largely mediated by interactions with transcription factors, modified histones, and methylated DNA and RNA (4). The tandem chromodomains of human CHD1 protein selectively bind to methylated lysine 4 on the histone H3 tail (H3K4) (33, 34), a hallmark of the transcriptionally active chromatin. The chromodomains target CHD1 to specific areas of chromatin-trimethylated H3K4 marks regions for open chromatin and transcriptional activation (35). Despite the double chromodomains of human CHD2 and yeast CHD1 share significant sequence similarity with human CHD1, they have much lower binding affinity to methylated H3K4 (33). In mice, the chromodomains of CHD1 are also required for proper chromatin localization (36). The SNF2-like ATP-dependent helicase domain shared in the CHD family anchors on the nucleosome and functions as an ATPase motor for the nucleosome remodeling (9, 36, 37). The SANT-SLIDE DNA-binding domains bind to DNA that flanks the nucleosome to increase the nucleosome-binding affinity of CHD1 and influence the direction of the nucleosome movement (12, 38).

CHD1 protein has DNA translocase activity that utilizes the energy of ATP hydrolysis to impel DNA around the octamer and mobilize nucleosomes (4). The CHD1 remodeler is a unique organization of domains on the nucleosome that reveals the direct interdomain communication (12, 37, 39). The chromodomains allow CHD1 to distinguish between nucleosomes and naked DNA by physically gating access to the ATPase motor (37). Disruption of the chromodomain-ATPase interface reduced the reliance on the histone H4 tail for nucleosome sliding (37). Besides, the chromodomains bind to nucleosomal DNA at the superhelical location (SHL) SHL1 site, resulting in ATPase closure; the ATPase motor binding to the SHL2 site is anchored to the N-terminal tail of histone H4 (12, 39). Both pack against the DNA-binding domain on DNA exiting the nucleosome (39). This arrangement spans and bridges two DNA gyres of the nucleosome and enables the ATPase motor to promote the translocation of DNA towards the nucleosome dyad, thereby loosening the first DNA gyre and remodeling the nucleosome (12, 39). The cycles of ATP hydrolysis of the ATPase motor trigger a succession of conformational changes of CHD1, promoting DNA translocation and nucleosome remodeling (4). By the endpoint of the remodeling reaction, the binding affinity of CHD1 for the nucleosome decreases, leading to its release from nucleosome substrates (40).

In addition to the assembly, disruption, and repositioning of nucleosomes, CHD1 is also involved in H3.3 histone variants incorporation and transcription regulation. H3.3 is deposited on gene bodies and regulatory elements marking active transcription, and its levels are constantly high throughout the cell cycle. In Drosophila models, depletion of CHD1 in embryos caused incorrect assembly of H3.3 in the paternal pronucleus chromatin, while CHD1 loss in the adult brain resulted in reduced H3.3 incorporation chromatin, global chromatin perturbation, transcriptional dysregulation, and metabolism reprogramming (41, 42). By disassembling nucleosomes at the promoter region, CHD1 promotes open chromatin and is associated with transcriptionally active locations throughout the genome (10, 43, 44). Deletion of *Chd1* resulted in the general downregulation of transcription by RNA polymerases I/II in mouse ESCs (45), and impaired efficient reprogramming of fibroblasts to the pluripotent stem cell state *via* downregulating the transcriptional factor Oct4 (10). Besides, CHD1 was also found to influence the pre-mRNA splicing, transcription initiation and transcription termination by bridging core factors to H3K4me3 (46–49).

Collectively, biochemical and structural studies reveal that CHD1 protein predominately interacts with methylated H3K4 histone marks, displays intricate conformational intradomain allosteric regulation, and exhibits nucleosome assembly and remodeling activities. This aligns with its epigenetic functions in chromatin organization, histone variants incorporation, and transcription reprogramming, and provides the mechanistic basis for understanding the phenotypes in animal models and human diseases with dysregulated CHD1.

## Dysregulation of CHD1 in human diseases

The tandem chromodomains of CHD1 are highly conserved among species. In yeast, the C-terminal is required for Chd1’s nucleosome-remodeling activity, and the combined mutations in the SANT domain (R1016/K1020) and SLIDE domain (R1255) abolish the binding of Chd1 to DNA and nucleosome and reduce its nucleosome-remodeling activity (50). In Drosophila, the Tryptophans W372/W375 mutants in the first chromodomain or W462 mutant in the second chromodomain impair CHD1’s interaction with trimethylation of H3K4 (H3K4me3) and reduce the assembly of H3.3 into chromatin (44). The flies containing these mutations have decreased viability and fertility (44).

Prior studies have demonstrated the key roles of CHD family remodelers in neurodevelopment in human being (10, 51–54). Large-scale exome sequencing in thousands of autism spectrum disorder cases identified recurrent *de novo* mutations in *CHD2* and *CHD8* as genuine autism risk factors (52–54). Pilarowski-Bjornsson syndrome is an autosomal dominant neurodevelopmental disorder characterized by delayed development and intellectual disability, often with autistic features, speech apraxia, and mild dysmorphic features. Several *de novo* heterozygous missense variants of *CHD1* (c.1853G>A, c.5123G>A, c.1379G>A, and c.421A>G) were identified in Pilarowski-Bjornsson syndrome and associated with the closed status of chromatin and the neurodevelopmental disability (51).

**Figure 2:**
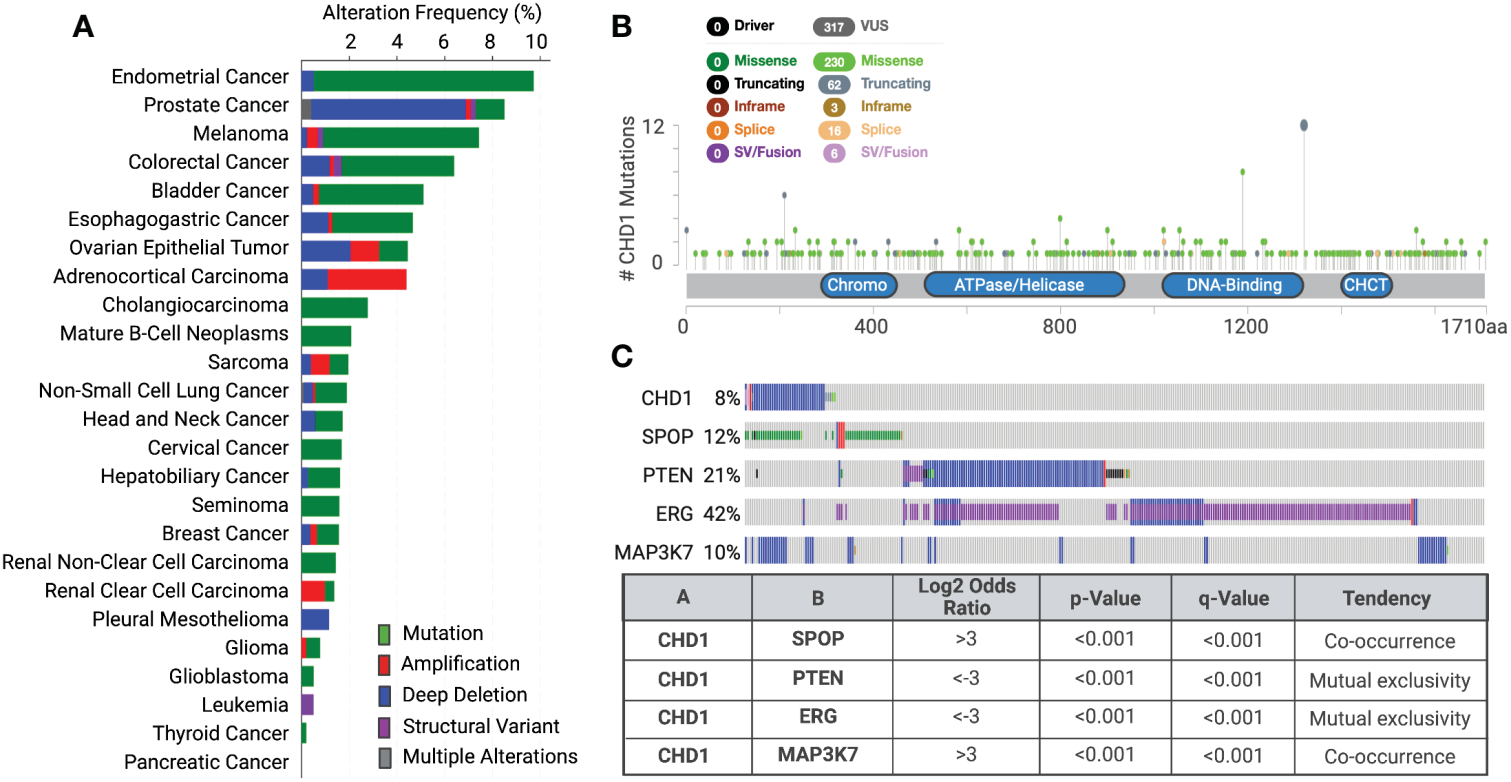
Genetic alterations of *CHD1* cross various cancer types **(A)** Genetic alterations of *CHD1* are frequent in human cancers (TCGA datasets). Different types of genetic alterations are highlighted in different colors. Their alteration frequency is presented in each cancer type. **(B)** Somatic mutations in CHD1 amino acid sequence across human cancers (TCGA databases). The number of single amino acid mutations is shown. Mutation diagram circles are colored with respect to the corresponding mutation types. The functional domains in the CHD1 protein are presented. **(C)** The co-occurrence and mutual exclusivity of CHD1 deletion and genetic alterations of SPOP, PTEN, ERG, and MAP3K7 in primary prostate tumors are shown (TCGA dataset). The Log2 Odds Ratio, p-value, q-value, and tendency between two genetic events are calculated (cBioportal).

Using genome sequencing techniques, many somatic mutations, copy number alterations, and chromosomal rearrangements of chromatin remodelers have been detected in the past decades. Recent cancer genomic studies identified recurrent mutations and deletions of the *CHD1* gene in prostate tumors (8-10%), uterine (11%), melanoma (7%), and colorectal cancers (6%) (**Figures 2A, B**) (55, 56). Mutations are more dominant than deletion in the *CHD1* gene in most cancer types, but not in prostate cancer. *CHD1* deletion was found in both localized prostate cancer and advanced castration-resistant prostate cancer (CRPC) (14–20). Recent epidemiology and genomics studies of prostate cancer in Asian men uncovered that *CHD1* is more often deleted (18%) in the East Asian population with localized prostate cancer than in Western patients (57, 58). Another recent study uncovered that subclonal deletion of *CHD1* is about three times more frequent in prostate tumors of African American (AA) men (29.7%) than that of European Ancestry (EA) men (11%) (59). Besides, CHD1 deletion is strongly associated with pathologic stages and rapid biochemical recurrence in AA cases (59).

Notably, deletions of *CHD1* show distinct patterns of co-occurrence and mutual exclusivity with genetic alterations of some oncogenes and tumor suppressor genes (**Figure 2C**). *CHD1* deletion often co-occurs with missense mutations in *SPOP* (speckle-type BTB/POZ protein) and defines a new molecular subtype of prostate cancer, characterized by increased DNA methylation and homogeneous gene expression patterns (60). Besides, *MAP3K7* and *CHD1* were significantly co-deleted in localized prostate tumors and combined loss correlated with poor disease-free survival of patients (20, 61). However, this co-occurrence is rarely found in other cancer types, suggesting their unique functions in prostate cancer development and progression. In contrast, *CHD1* deletion is mutually exclusive with *PTEN* loss or *TMPRSS2:ERG* fusion in human prostate tumors (14, 16, 25, 62), by crosstalk with key components in PTEN-AKT and AR signaling pathways.

The expression of CHD1 is modulated at both post-transcriptional and post-translational levels, and its dysregulation is associated with cancer development and other human diseases (**Table 1**). MicroRNAs (miRs) represent a critical class of small, non-coding RNAs and repress target genes either by mRNA degradation or repression of translation. Lifespan-related miRNAs, miR-34a, miR-107, and miR-212-3p, are found preferentially target *Chd1* and are associated with high-fat diet and aging (63). In estrogen receptor (ER)+ breast cancer, miR-26 is identified as a microRNA targeting *CHD1* and suppresses breast cancer cell proliferation by downregulating the CHD1 expression (67).

**Table 1:** Dysregulation of CHD1 in human diseases.

Type	Dysregulation	Mechanism	Diseases
**Genetic** **Alterations**	*CHD1* Deletion	* Alter AR transcriptome * Chromatin instability * Defects in DNA damage repair * Lineage Plasticity	Prostate cancer (14–20)
	Missense/Truncating Mutations of *CHD1*	* To be determined	Uterine, melanoma, colon, and other cancers (55, 56)
	Missense Mutations of *CHD1*	* Dysregulated chromatin * Neurodevelopmental disability	Pilarowski-Bjornsson syndrome (51)
**Epigenetic-** **MicroRNA Targeting CHD1**	High expression of miR-34a, miR-107, miR-212-3p	* Down-regulate CHD1 expression * Mimic High-fat diet and aging-induced transcriptome * Activation of transposons	Metabolic diseases Aging (63)
	Repressed expression of miR-26	* Repression of miR-26 causes CHD1 up-regulation * CHD1 is required for estrogen-induced cell growth upon miR-26 depletion	ER+ breast cancer (56)
**Disrupted Post-translational Modification**	Disrupted ubiquitination and proteolysis of CHD1	* PTEN-AKT-GSK3β signaling promotes CHD1 proteolysis *via* the β-TrCP-mediated ubiquitination-proteasome pathway * Stabilization of CHD1 protein promotes tumor progression in PTEN-deficient tumor	PTEN-deficient prostate and breast cancer (25–27)
	Hyper-SUMOylation of CHD1 protein	* SUMO E2 ligase Ubc9 sustains the transformation growth of KRAS-mutated colorectal cancer cells * CHD1 is hyper-SUMOylated by UBC9 and mediates the KRAS-driven transformation	KRAS-mutated colorectal cancer (64, 65)
	Increased SUMOylation of CHD1	* Influenza virus induces SUMOylation of CHD1 and other proteins involved in RNA polymerase II transcription and chromatin remodeling	Influenza virus infection (66)

Our prior studies in prostate cancer demonstrated that PTEN-AKT-GSK3β signaling promotes CHD1 protein degradation *via* the β-TrCP-mediated ubiquitination-proteasome pathway (25). β-TrCP is an F-box protein that acts as the substrate-recognition subunit for the SCF^β-TrCP^ (Skp1–Cullin1–F-box protein) E3 ubiquitin ligases. We found that β-TrCP E3 ligase directly interacts with CHD1 protein, induces its poly-ubiquitination, and promotes the proteolysis of CHD1 (25). Through E3 ligase consensus-sequence scanning, we also identified two evolutionarily conserved putative β-TrCP consensus-binding motifs (DSGXXS) at the N terminus of CHD1 (25). Another study also reported that the N-terminal serine-rich region (SRR) of CHD1 is modified by phosphorylation and depletion of SRR impaired differentiation of the ESCs (68). Systematic mass spectrometric analysis and consensus site prediction also showed that PGK and GSK3 kinases might be involved in the phosphorylation of CHD1 (69). Notably, β-TrCP E3 ligase recognizes and interacts specifically with phosphorylated substrates, and importantly, β-TrCP-binding motifs in CHD1 protein contain GSK3β consensus sequences (SXXXS). Further biochemical and molecular biological studies established that GSK3β serves as a kinase of CHD1 and mediates its recognition and interaction with β-TrCP E3 ligase, resulting in CHD1 protein ubiquitination and degradation (25). In PTEN-deficient cancers, AKT activation-induced GSK3β suppression results in the disruption of CHD1 proteolysis and aberrant accumulation of the CHD1 protein (25–27), which contributes to tumor development and tumor microenvironment (TME) remodeling.

Like ubiquitination, SUMOylation is a post-translational modification that regulates protein stability, activity, localization, and interactome. SUMOylation involves various cellular processes, such as transcription, chromatin remodeling, DNA damage repair, cell cycle progression, ribosome biogenesis, and mitochondrial dynamics (70–72). In KRAS-mutated colorectal cancer, CHD1 protein is hyper-SUMOylated by the SUMO E2 ligase UBC9, and depletion of CHD1 impairs the KRAS-driven transformation (64, 65). Besides, influenza virus infection was also found to induce the SUMOylation of CHD1 and other proteins involved in RNA polymerase II transcription and chromatin remodeling (66).

As a chromatin remodeler, CHD1 dysregulation is associated with malignancies and other human diseases. Diverse mechanisms, including genetic alterations, epigenetic regulations, and post-translational modifications, lead to the dysregulation of CHD1 in context-dependent manners (**Table 1**). It is equally important to understand the biological functions of CHD1 in different contexts, which will uncover the therapeutic vulnerabilities of diseases with dysregulated CHD1.

## Multifaceted roles of CHD1 in prostate cancer

Genetic studies in yeast, fruit flies, zebrafish, and mice underscore the roles of CHD family enzymes in regulating cellular fate and identity, embryonic development, stem cell maintenance, and neuronal development and pathologies. These studies have been summarized and discussed in several comprehensive review articles (2, 4, 5). The increasing evidence documented individual CHD remodelers function as context-dependent oncogenes or tumor suppressors in human malignancies. For instance, CHD4, as a crucial subunit of the NuRD complex, promotes tumorigenesis by epigenetic silencing tumor suppressor genes or serving as a coactivator of HIF in colorectal, breast, and endometrial cancers (73–75). In contrast, CHD5 was identified as a tumor suppressor gene in gliomas, breast, colon, lung, ovarian, and prostate cancers (76, 77). Given the frequent alterations and dysregulation of CHD1 in prostate tumors, in this section, we review CHD1’s biological functions in prostate cancer with an emphasis on its crosstalk with different genetic alterations and diverse signaling pathways.

### Prostate tumorigenesis

As noted earlier, *CHD1* is homozygously deleted in 8~18% of prostate cancer, supporting the hypothesis that *CHD1* is a tumor suppressor in prostate cancer. Earlier *in vitro* studies using siRNA showed that downregulation of *CHD1* in nontumorigenic prostate epithelial cells promoted cell invasiveness and enhanced cell clonogenicity, but had no impact on cell growth (17, 18). To obtain the genetic evidence, our and other independent groups established prostate-specific *Chd1* deletion genetically engineered mouse (GEM) models, in which conditional *Chd1* alleles deleted by a Probasin (Pb) promoter-driven Cre recombinase (*Pb-Cre; Chd1^L/L^*) (21, 23, 27). Homozygous deletion of *Chd1* in prostate glands showed no observed differences in cell proliferation, cell survival, androgen receptor (AR) expression, or glandular structure (21, 23, 27). No invasive adenocarcinoma was observed in mice up to 1 year of age, as characterized by well-maintained smooth muscle actin structures (21). This genetic evidence suggests that *Chd1* loss alone is insufficient to drive tumorigenesis in the prostate.

**Figure 3:**
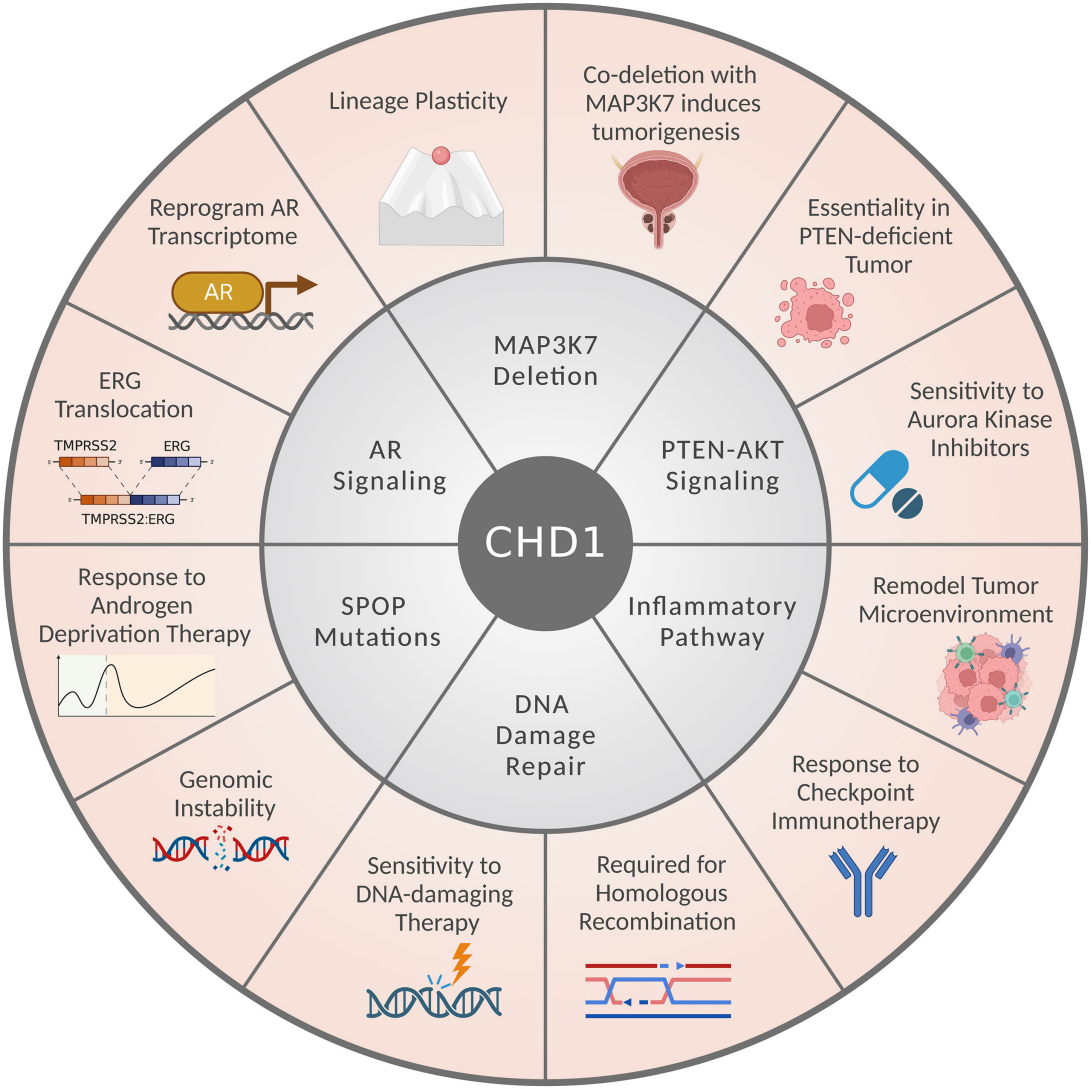
CHD1’s multifaceted roles and crosstalk with signaling pathways in prostate cancer. The CHD1-associated signaling pathways or genetic alterations are presented in the middle circle. CHD1’s biological functions and therapeutic implications in prostate cancers are listed in the outer circle.

Notably, CHD1-depleted tumors often harbor additional genetic alterations, including *SPOP* mutations and *MAP3K7* deletion, but also show mutual exclusivity with *PTEN* loss or *ERG* translocation (**Figure 2C**) (14, 16, 17, 20, 25, 26, 62). CHD1 depletion reduced cell proliferation, invasiveness, and tumor growth of PTEN-deficient cancer cells (14, 20, 25, 26); while loss of *MAP3K7* and *CHD1* coordinates to promote aggressive prostate cancer (20, 61). These observations seem paradoxical at first glance; however, they established the context-dependent roles of CHD1 in prostate cancer (**Figure 3**). Importantly, CHD1’s distinct roles in different contexts are largely mediated by the crosstalk with diverse signaling pathways, which will be introduced individually in the following subsections.

### AR signaling

Prostate cancer is largely driven by androgen receptor (AR) signaling. Androgen deprivation therapy (ADT) and AR inhibition are the main strategies for prostate cancer treatment (78). Although CHD1 protein does not directly bind to AR (14, 16, 23), loss of *CHD1* caused the transcriptome reprogramming of AR signaling and is strongly associated with the ERG translocation (14, 16, 22, 23). By performing the chromatin-bound interactome analysis, Augello et al. uncovered that CHD1 interacts with the cofactors of AR and other nuclear receptors (23). Chromatin immunoprecipitation (ChIP) sequencing showed that CHD1 colocalizes to gene enhancers enriched for AR and its cofactors, such as HOXB13, ETV1, and FOXA1 (23). Specifically, they found that CHD1 localizes to chromatin-containing canonical AR binding sites, but *CHD1* loss causes AR to redistribute to HOXB13-enriched sites, which drives a unique AR transcriptome that contributes to the tumor formation (23).

In prostate cancer, the most common genetic rearrangement involves the fusion of the androgen-regulated gene *TMPRSS2* with the ETS transcription factor ERG (79). The fusion joins the 5′-UTR of *TMPRSS2* (21q22) with the 3′-end of *ERG* (21q22) and leads to the TMPRSS2:ERG mRNA fusion transcript, which is induced by androgen. Using whole exome sequencing, FISH, or confocal microscopy, several groups showed the mutual exclusivity of CHD1 deletion with ERG fusion in human prostate tumors (14, 16, 62). CHD1 deletion is also strongly associated with early PSA recurrence (14, 59). Using a doxorubicin/dihydrotestosterone-induced DNA double-strand breaks system, Burkhardt and colleagues showed that CHD1 depletion prevents the formation of ERG rearrangements. Mechanistically, they found that CHD1 is required to recruit AR to responsive promoters and regulates the expression of AR-responsive genes, such as NKX3-1, FOXO1, and PPARγ (14). Given that AR-dependent transcription is a prerequisite for ERG translocation, these studies concluded that a functional CHD1 supports AR signaling transcriptome and ERG fusion development in prostate cancer.

Lysine-specific demethylase 1 (KDM1A/LSD1) removes the mono- and di-methylation from H3K4 and H3K9, and plays an important role in regulating AR-dependent gene expression in prostate cancer (80, 81). A prior study by the Schule group reported that the LSD1 protein is modified by di-methylation at K114 (K114me2) (82). By solving the cocrystal structure, they identified CHD1 as an LSD1-K114me2 reader and uncovered that chromatin colocalization of CHD1 and LSD1-K114me2 drive AR-dependent transcription and TMPRSS2-ERG translocation (82). This structural study provides additional evidence and mechanistic insight into CHD1’s roles in modulating AR signaling and ERG fusions during prostate cancer evolution.

### Lineage plasticity

Transcriptomic and epigenetic profiling studies in ESCs and cancer cells showed that CHD1 is required for sustaining the opening of chromatin and the global transcription (10, 22, 23, 25, 27, 83). CHD1 deficiency causes the accumulation of heterochromatin, diminishing the pluripotency of ESCs (10). In prostate cancer, CHD1 co-localizes with H3K4me3 to the promoters of actively transcribed genes, while CHD1 depletion reduces H3K4me3 marked genes, alters the chromatin assembly across the genome, and reprograms the global transcription (22, 23, 25). Lineage plasticity of cancer cells has been proposed as a source of intratumoral heterogeneity and resistance to targeted anticancer treatments (84). In prostate cancer, the histological transformation from AR-dependent adenocarcinoma to AR-indifferent neuroendocrine or small-cell carcinoma is a well-known pathway of lineage plasticity, which might occur as a consequence of ADT (85, 86). In addition to the deregulation of AR signaling, *CHD1* loss is linked to lineage plasticity by inducing a lineage-specific transcriptome (20, 22).

Cramer’s group initially proposed this hypothesis. They found *MAP3K7* and *CHD1* were significantly co-deleted in localized prostate tumors and combined loss correlated with poor disease-free survival of patients (20, 61). CHD1 knockdown reduced cell proliferation, impaired tumor growth, and prolonged the overall survival of mice in PTEN-deficient LNCaP-derived xenograft models. However, additional *MAP3K7* loss completely rescued this effect and promoted prostate cancer progression (20, 61). Co-suppression of MAP3K7 and CHD1 induces androgen-independent growth and causes resistance to AR inhibitors, such as enzalutamide (61). Combining mouse prostate epithelial progenitor/stem cells (PrP/SC) and tissue recombination model, they found that CHD1-depleted PrP/SCs grafts are mostly benign, characterized by intact p63+ basal layer (20). This is consistent with the phenotypes observed in *Pb-Cre; Chd1^L/L^* GEM model (21, 23, 27). In contrast, MAP3K7-depleted grafts displayed a mixture of benign, high-grade prostatic intraepithelial neoplasia (PIN), and carcinoma phenotypes. Strikingly, dual *MAP3K7*–*CHD1* loss grafts displayed high-grade PIN and invasive carcinoma phenotypes (20). Compared to MAP3K7 or CHD1 depletion alone, dual depletion caused lineage switching, characterized by loss of AR and epithelial markers (CK5, p63, CK14, and CK18) along with the upregulation of neuroendocrine differentiation markers (SYP and Nestin) and mucin production (20). It remains unclear if *MAP3K7*/*CHD1* double-depletion affects metastatic progression. Nevertheless, better understanding their interactions and underlying mechanisms might provide novel therapeutic strategies for *MAP3K7*/*CHD1* loss prostate cancer.

Recently, Zhang et al. showed that *CHD1* loss renders prostate cancer cells more resistant to AR inhibition *via* inducing lineage plasticity (22). They showed that loss of *CHD1* induces the transcription factors of GR, BRN2, TBX2, and NR2F1, which are required to promote tumor heterogeneity and resistance to AR inhibitors in CHD1-deficient tumors (22). They also found that enzalutamide-resistant xenograft tumors with CHD1 depletion and high expression of those transcription factors, lost luminal lineage identities (AR, CK8, and CK18), but displayed increased basal markers (CK5 and p63) and epithelial to mesenchymal transition genes (SNAI2, TWIST1, SNAI1, and ZEB1) (22). These non-luminal lineage programs and plastic chromatin landscape induced by *CHD1* loss may serve as mechanisms to enable heterogeneous subclones less dependent on AR.

### DNA damage repair

Endogenous cell metabolism and environmental factors often cause DNA double-strand break (DSB). Homologous recombination (HR) and non-homologous end joining (NHEJ) are two major repair mechanisms in response to DSB (87). Using prostate cancer cell lines and GEM models, several studies reported that *CHD1* loss causes defects in HR-mediated DNA damage repair (DDR) and increases sensitivity to DNA-damaging therapies (21, 24, 88, 89). Besides, recent studies in metastatic prostate cancer patients showed that CHD1 deletion is associated with HR deficiency-related mutational signatures (59, 90).

Mechanistically, CHD1 accumulates at the DNA damage sites, maintains the open status of chromatin, and co-localizes with γH2AX in response to DNA damage (24). CHD1 interacts with and recruits DDR factors, such as CtIP, 53BP1, RIF1, and KU70, to the DNA damage sites (21, 24). CtIP is a key player in HR by resecting DSB ends. *CHD1* loss impairs the recruitment of CtIP to DNA damage sites and suppresses the initiation of HR (24, 88). As a key DDR protein, 53BP1 maintains the balance of repair pathway choices and genomic stability. Shenoy et al. found that CHD1 forms a complex with NHEJ components and negatively regulates the protein stability of 53BP1 (21). *CHD1* loss stabilizes 53BP1 protein and causes the switch from HR to NHEJ pathway for DSB repair. Although AR signaling is known to regulate the expression of DDR-related genes and promotes NHEJ repair, the role of CHD1 in modulating DDR is independent of the AR pathway (21). *CHD1* loss is also associated with chromosomal and genomic instability in prostate cancers (21, 22), and DDR defects may serve as one of the mechanisms.

### When CHD1 loss meets SPOP mutations

Recurrent missense mutations in *SPOP* (speckle-type BTB/POZ protein) occur in 10-15% of localized prostate tumors and metastatic CRPC (60, 91–93). In occurrence with CHD1 deletion (**Figure 2C**)*, SPOP* mutations define a distinct prostate cancer subtype, characterized by genomic instability, increased AR transcriptional activity, absence of ERG rearrangements, and increased DNA methylation (60, 91, 92, 94). SPOP protein is a substrate adaptor for the Cullin3-RING-based BCR E3 ligase complex (CUL3-SPOP), which mediates the ubiquitination and proteasomal degradation of target proteins. In prostate cancer, hotspot *SPOP* mutations are only observed in the MATH domain that is responsible for substrate recognition and recruitment. The mutant reduces the substrate-binding affinity and results in the aberrant accumulation of substrates (95).

Several oncogenic proteins in AR signaling were identified as substrates of CUL3-SPOP, such as AR (96), SRC3 (97), and ERG (98, 99). CUL3-SPOP complex mediates the ubiquitination-degradation of AR by binding to the ^645^ASSTT^649^ Motif in the hinge domain of AR. Prostate cancer-associated SPOP mutants (Y87C, Y87N, F102C, S119N, F125V, W131G, F133L, and F133V) fail to bind AR protein, thereby increasing the protein stability and activity of AR during tumorigenesis (96, 100). By establishing a tissue-specific SPOP-F133V overexpressing GEM model, Blattner and colleagues reported that *SPOP* mutation promotes prostate tumorigenesis through coordinate regulation of PI3K/mTOR and AR signaling (101). Clinical trials in men with metastatic prostate cancer found that SPOP mutations are associated with improved survival outcomes after ADT (93, 102, 103). Although it remains unclear whether *SPOP* mutations crosstalk with *CHD1* loss when regulating AR signaling, a clinical study in metastatic CRPC showed that *SPOP* mutations and *CHD1* loss are associated with a higher response rate to abiraterone (inhibitor of androgen biosynthesis) and a longer time on the abiraterone treatment (93).

In addition to modulating AR signaling, coordinate *CHD1* deletion and *SPOP* mutations are also involved in DNA damage response. Phenocopying CHD1 loss, SPOP mutations also cause genomic instability and impaired HR DSB repair, as well as promote the sensitivity of prostate tumors to DNA-damaging therapeutic agents, such as PARP inhibitors (94, 104, 105). Mechanistically, SPOP is accumulated at DNA double-strand break sites, where it interacts with ATM kinase and plays an essential for DDR (94, 105). Depletion or mutations of SPOP inhibits HR and promotes NHEJ by downregulating DNA repair factors (RAD51, BRCA2, CHK1, and ATR), reducing RAD51 foci formation, and stabilizing 53BP1 (94, 106). Recent studies found that SPOP mutations and CHD1 deletion sensitize prostate cancer cells to DNA damage inducers and show synergistic effects on the DNA damage repair (59, 89). By generating prostate-specific *Chd1* and/or *Spop* deletion GEM models, Zhu and colleagues found that co-deletion of *Chd1* and *Spop* in the prostate synergistically induces the response to DNA DSBs, characterized by increased γH2AX staining (89). Besides, they showed that the combination of *CHD1* depletion and *SPOP* mutations significantly augmented the DNA damage response and sensitized human prostate cells to DNA-damaging agents (89). Another study in AA men revealed that, compared to cases with either alteration alone, prostate tumors with both *CHD1* deletion and *SPOP* mutations showed significantly higher levels of HR deficiency-associated signatures and large-scale structural rearrangements (59). These studies demonstrated the synergistic effects of *CHD1* loss and *SPOP* mutations in modulating AR signaling and DDR pathways, providing insights into the molecular basis of their frequent co-occurrence in prostate cancers.

### Essentiality in PTEN-deficient cancers

Tumor suppressor *PTEN* is frequently altered in prostate and other cancer types. As a dual lipid and protein phosphatase, PTEN dephosphorylates PIP3 and suppresses the activation of AKT, leading to a hyperactive PI3K signaling (107, 108). PTEN/AKT pathway is critical for cellular processes, such as metabolism and cell proliferation (109). Genetic deletion and mutations of *PTEN* occur in ~20% of localized prostate tumors and are further enriched in ~40% of CRPC with strong associations with metastatic disease and poor overall outcome (60, 110). In prior studies, we found that *CHD1* deletions show a mutually exclusive pattern with *PTEN* loss in prostate tumors (**Figure 2C**), and CHD1 negatively correlates with PTEN expression at protein levels (25). Mechanistically, *PTEN* loss stabilizes CHD1 protein in cancer cells and prostate tumors by disrupting CHD1’s ubiquitination and degradation (25–27), as described above. Functionally, we identified CHD1 as a synthetic essential gene in cancers containing PTEN deficiency (25–27). *CHD1* depletion significantly suppressed tumor growth in PTEN-deficient xenograft models (25), consistent with earlier observations in LNCaP xenograft tumors (20). However, *CHD1* knockdown showed little effect on benign prostatic hyperplasia cells or PTEN-intact tumors (25, 26).

In GEM models, Pb-Cre-driven *Pten* loss (*Pb-Cre; Pten^L/L^*) in the prostate triggers non-lethal invasive tumors after a long latency (111). By crossing a *Chd1* conditional knockout allele into this GEM model, Augello et al. reported that *CHD1* loss promotes prostate tumor progression (23). The limitations of this study include the small animal cohort (n = 5), low frequency of tumor progression (1 in 5 mice), and lack of survival data. In contrast, we established prostate-specific *Chd1* deletion in two well-established PTEN-deficient GEM models, *Pb-Cre; Pten^L/L^* and *Pb-Cre; Pten^L/L^; Smad4^L/L^* (112), and then determined the impact of *Chd1* deletion with much larger cohorts (n = 22 or 18) (27). In both models, we found that CHD1 depletion significantly delayed the development and progression of PTEN-deficient prostate tumors and prolonged the survival of mice, providing genetic evidence supporting the essential roles of CHD1 in the context of PTEN defects (27). Given that CHD1-null prostates are phenotypically normal (21, 23, 27), these studies revealed the therapeutic potential of targeting CHD1 in PTEN-deficient tumors with an acceptable therapeutic window. Despite these encouraging factors, it is worth noting that tumor progression was rarely observed in some Pten/Chd1 double-knockout mice. Although these cases appear to result from clonal expansion of prostate cancer cells undergoing incomplete Chd1 deletion, future study is needed to identify potential second-site suppression events that may underlie CHD1 bypass. It will also provide rational combinatorial strategies targeting CHD1 in PTEN-deficient tumors.

### Tumor microenvironment remodeling

Tumor development and progression are largely driven by interactions between cancer cells, extracellular matrix, stromal cells, and immune cells in the tumor microenvironment (TME) (78). Prostate cancer has a TME characterized in part by a relative paucity of infiltrating T cells and a high proportion of immunosuppressive myeloid cells, including myeloid-derived suppressor cells (MDSCs) and tumor-associated macrophages (TAMs) (78, 113). MDSCs are a heterogeneous group of myeloid cells that play immunosuppressive roles *via* interaction with T and NK cells (114). Our prior studies using multiple GEM models demonstrated that CHD1 is involved in the inflammatory response and plays a crucial role in modulating the TME *via* promoting MDSC infiltration and suppressing tumor-infiltrating lymphocytes (TILs) (27). In PTEN-deficient prostate tumors, *CHD1* deletion caused reduced MDSC infiltration and increased CD8+ T cells (27). Transcriptional and epigenetic profiling analyses revealed that CHD1 cooperates with NF-κB, the central player of inflammation, to regulate the transcription of inflammatory genes (25, 27). Besides, we identified IL-6 as a direct target of CHD1 and mediates the recruitment and activation of MDSC, which contributes to T cell suppression in the prostate tumors (27). In addition to NF-κB and IL-6/Stat3 signaling, CHD1 modulates several other TME-related pathways, such as inflammatory response, interferon alpha and gamma pathways, and angiogenesis (23, 27).

A recent immunogenicity study in localized prostate cancer provides additional evidence. Using multiplex immunofluorescence, Calagua and colleagues identified the genomic alterations associated with immunogenic (PD-L1 ≥5% and extensive TILs) and nonimmunogenic (PD-L1 negative and no TILs) tumor foci (115). They found that deep deletions of *CHD1* are strongly associated with dendritic cell signatures and immunogenic phenotype, characterized by enriched T cell infiltration (115). The regulatory axis of CHD1/IL-6/MDSC may serve as one mechanism by which *CHD1* loss drives immunogenicity. Besides, immunogenic localized prostate cancer shows high rates of genomic instability and variable tumor mutational burden (TMB) (115), suggesting chromatin instability and DDR defects induced by *CHD1* loss may also contribute to immunogenic features.

## Therapeutic strategies targeting CHD1 dysregulation

In the past decade, we have gained a better understanding of CHD1 biology and how its dysregulation impacts cancer development and progression. This knowledge lays an important foundation for developing effective therapeutics targeting the dysregulated CHD1 in cancers and using CHD1 as a biomarker for predicting the response to therapies. In this section, we highlight the response of CHD1-deficient tumors to DNA-damaging and antiandrogen therapies. Given that CHD1 is upregulated and plays an essential role in PTEN-deficient cancers, we also discuss the therapeutic potential of targeting CHD1 and its downstream effectors in tumors containing PTEN deficiency.

### DNA-damaging therapy

As noted above, CHD1 plays a key role in DNA damage response and modulates the choice between HR and NHEJ DDR pathways. Several preclinical studies using prostate cancer cell lines, PDX models, and GEM models demonstrated that *CHD1* loss leads to hypersensitivity to ionizing radiation (IR), PARP inhibition, and DNA-damaging agents, such as mitomycin C, carboplatin, irinotecan, and camptothecin (21, 24, 88–90).

By comparing the response of wildtype and *Chd1*-null (*Pb-Cre; Chd1^L/L^*) mice to a single dose of 10 Gy of IR, Shenoy and colleagues found that *Chd1* deleted prostate tissues and ESCs are more sensitive to IR, as evidenced by increased γH2AX and phosphorylation of H2A and p53 (21). Similar phenotypes were also observed in *CHD1*-depleted prostate cell lines (21, 24). PARP (Poly-ADP-ribose polymerase) detects and initiates single-strand DNA breaks (SSB) DNA damage repair. Prior studies uncovered that PARP inhibitors have synthetic lethal effects in cells with HR defects, such as *BRCA1* and *BRCA2* loss (116). PARP inhibitors have been clinically tested in CRPC, and genetic alterations in DDR pathways are associated with better responses (117, 118). Preclinical studies showed that *CHD1* loss-induced HR defects sensitize prostate tumors to PARP inhibitors, Olaparib and Talazoparib, both *in vitro* and *in vivo* (21, 24, 59, 88), suggesting CHD1 might be a predictive biomarker. The second-generation platinum agent, carboplatin, also showed a good response in a metastatic CRPC patient with homozygous *CHD1* loss (21).

Notably, *SPOP* depletion also sensitizes cancer cells to IR and PARP inhibitors (94, 104, 105). A recent study demonstrated that *SPOP* mutations and *CHD1* loss synergistically promote sensitivity to camptothecin, an inducer of double-strand breaks (89). Given that co-occurrence of *SPOP* mutations and *CHD1* deletion define a distinct molecular subtype of prostate cancer, further studies are needed to assess if they have synergistic effects in response to DNA-damaging therapies. Their potential as biomarkers for predicting the response to radiotherapy, PARP inhibitors, and DNA-damaging agents in advanced prostate cancers remains to be determined.

### Antiandrogen therapy

In 1941, Huggins and Hodges reported that castration led to tumor regression in prostate cancer patients, first recognizing hormone responsiveness as a central feature of prostate cancer (119). Androgen deprivation by castration or agents that block the androgen pathway is the standard of care for prostate cancer. Resistance to ADT facilitates the development of CRPC with high rates of metastasis and mortality (120). Given the important role of CHD1 in AR signaling, preclinical and clinical studies have been conducted to determine the impact of *CHD1* loss on response to antiandrogen therapies using different model systems (14, 16, 22, 23, 61).

Using an androgen-driven regrowth model, Augello et al. showed castrated Chd1-deficient mice (*Pb-Cre; Chd1^L/L^*) showed increased proliferation in regenerated epithelium upon androgen re-stimulation, suggesting *Chd1* deletion may render the prostate tissue more dependent to androgen (23). However, Zhang et al. used AR-overexpressing LNCaP models and showed that *CHD1* loss renders human prostate cancer cells more resistant to AR inhibitors *in vitro* and *in vivo* in castrated mice (22). They also found that low expression of CHD1 is associated with shorter clinical response to next-generation antiandrogen therapies (enzalutamide or abiraterone) in CRPC patients (22). Along the same line, Jillson and colleagues showed that co-suppression of MAP3K7 and CHD1 causes androgen-independent growth of prostate cancer cells and promotes resistance to AR inhibitor enzalutamide (61).

In prostate cancer patients, *CHD1* loss was associated with a shorter time to PSA recurrence, suggesting its potential as a prognostic biomarker (14, 59, 61, 121). However, recent clinical trials in men with metastatic prostate cancer found that *SPOP* mutations are associated with improved survival outcomes after ADT (93, 102, 103). When considered as an individual variable, *CHD1* loss is associated with a higher response rate to abiraterone (OR, 7.30, *P*= 0.08) and a longer time on abiraterone (HR, 0.50, *P* = 0.06) in metastatic CRPC patients (93). Prospective clinical trials are needed to validate the impact of *CHD1* deletion on response to castration, abiraterone, enzalutamide, and other antiandrogen drugs in both hormone-sensitive and -resistant prostate cancers. Given the context-dependent role of CHD1 in prostate tumors, genes showing co-occurrence (*SPOP* or *MAP3K7*) or mutual exclusivity (*ERG* and *PTEN*) should also be considered as influence factors in these clinical studies.

Notably, the upregulation of transcription factors of GR, BRN2, TBX2, and NR2F1 was found to mediate the resistance to enzalutamide in *CHD1*-deficient prostate cancer, since inhibition of each factor re-sensitizes *CHD1* loss prostate tumors to AR inhibitor (22). This offers new insights into synthetic lethal interactions with CHD1 and potential therapeutic vulnerabilities in prostate cancers containing CHD1 deficiency. Given that GR (Glucocorticoid Receptor) inhibition has been tested in clinical studies of CRPC (NCT02012296), future biomarker studies are needed to assess if GR inhibition is more effective in CRPC patients harboring *CHD1* loss.

### Targeting CHD1 in PTEN-deficient cancers

Our prior studies in xenograft and GEMM models established CHD1 as a synthetic essential gene and potential therapeutic target in prostate cancers containing PTEN defects (25–27). Several independent groups are dedicated to developing small-molecule inhibitors targeting CHD1, and the efficacies of top hits will be tested in cancer cell lines and diverse preclinical models. We expect that these drugs have better therapeutic effects on PTEN-deficient tumors but may have modest effects on PTEN-intact tumors. When some of them enter the early clinical phase, it is important to use PTEN as a biomarker for patient selection. Given that CHD1 inhibition sensitizes tumor cells to DNA-damaging agents, the combination of CHD1 inhibitors and DNA-damaging therapies should be tested in preclinical and clinical studies as well. It is also worth determining if CHD1 inhibitors synergize with AR or GR inhibitors in suppressing CRPC tumor growth and progression. However, caution should be taken when pharmacologically inhibiting CHD1 in prostate cancer with *SPOP* or *MAP3K7* deletions, reasoning that CHD1 inhibition may play tumor-promoting roles in these contexts.

### Aurora kinase inhibitors

Combining high-throughput epigenetic screening and pan-cancer drug sensitivity analyses, we reported that CHD1 promotes the susceptibility of cancer cells to inhibitors targeting Aurora kinases (26). Aurora kinases are key players in mitotic control. Among three mammalian paralogues, Aurora A is required for centrosome maturation and mitotic spindle assembly (122–124). Several small-molecule inhibitors targeting Aurora kinases have been tested in clinical trials, and subsets of patients showed significant clinical benefits from the single agent or in combination with other agents (125–131).

In our recent study, we uncovered that *CHD1* loss impaired the *in vitro* and *in vivo* efficacy of Aurora kinase inhibitors, while high expression of CHD1 is associated with increased sensitivity in a pan-cancer manner (26). Prior studies demonstrated that the activity of Aurora A is largely modulated by the autophosphorylation and interaction with the co-activator TPX2 (132–135). Mechanistic studies revealed that the regulatory axis of CHD1-KPNA2 suppressed the interaction between Aurora A and TPX2, thereby rendering cancer cells more vulnerable to Aurora A inhibition (26). Furthermore, our studies in GEM models, patient-derived organoids, and patient samples showed that PTEN defects are associated with a better response to Aurora A inhibition in advanced prostate cancer by inducing CHD1 protein stabilization (26). This study establishes the important role of CHD1 in modulating Aurora kinases and provides insights for using PTEN and CHD1 as predictive biomarkers to improve patient selections in clinical trials of Aurora A inhibitors.

### Checkpoint immunotherapy

Immunotherapy has shown only modest activity in advanced prostate cancer, partially due to low tumor mutation burden (TMB), lack of infiltrating T cells, and immunosuppressive TME (78, 113). Immune checkpoint inhibitors that target cytotoxic T-lymphocyte-associated protein 4 (CTLA-4), programmed death 1 (PD-1), and its ligand (PD-L1) display minimal or no activity as single agents or in combination with AR inhibitors in advanced prostate cancers (136–141). As noted above, CHD1 contributes to immunosuppressive TME by promoting MDSCs and suppressing tumor-killing T cells (27). Our recent studies in GEM and syngeneic models revealed that depletion of CHD1 reverses the immunosuppressive TME and sensitizes prostate tumors to the checkpoint immunotherapy (27). As a direct target gene of CHD1, IL-6 mediates the recruitment and activation of MDSCs in prostate tumors. Phenocopying CHD1 depletion, pharmacological inhibition of IL-6 and dual blockade of PD-1/CTLA-4 showed synergistic effects in preclinical models of PTEN-deficient prostate cancer (27). Notably, IL-6 inhibition was found to reduce immune-related adverse events in patients by de-coupling autoimmunity from antitumor immunity induced by immune checkpoint blockade (142). Further clinical studies are needed to test the above combinations in CRPC patients, particularly in PTEN-loss/CHD1-high tumors.

## Conclusion and perspective

CHD1 was discovered over two decades ago, and significant progress has been made in understanding CHD1 biology. However, many questions remain to be answered, regarding CHD1’s context-dependent roles and the molecular basis in human diseases, as well as the modeling systems for studying CHD1 biology and therapeutics development.

It remains a debate on whether CHD1 is a tumor suppressor or an oncogene during tumorigenesis and cancer progression. Prior studies in cell lines and GEM models showed that *CHD1* deletion alone is insufficient to drive prostate tumorigenesis (21, 23, 27). Functionally, CHD1 is required for conventional AR signaling and transcriptome (14, 16, 22, 23), which plays a key role in prostate cancer development and progression. However, *CHD1* loss causes chromatin instability and lineage plasticity, resulting in the androgen-independent growth of prostate tumors and less sensitivity to antiandrogen therapy (14, 16, 22, 23, 61). As discussed above, the impact of *CHD1* loss may vary when combined with different genetic alterations in prostate cancer. In the context of PTEN deficiency, CHD1 is essential for tumor growth and the immunosuppressive TME (25, 27); in contrast, *CHD1* deletion augments the tumor-promoting role of *MAP3K7* loss (20, 61). Hence, through interacting with different genetic events and altering the transcription of distinct pathways, CHD1 possesses the capability to exert both oncogenic and tumor-suppressive functions in context-dependent manners.

Most CHD family members are components of large multi-subunit complexes, however, CHD1 remodeler exists predominantly as a monomer or dimer (9, 143). The epigenetic machinery and interactome of CHD1 have been reported in different species. The yeast Chd1 was identified as a component of SAGA (Spt-Ada-Gcn5 acetyltransferase) and SLIK (SAGA-like) complexes, two highly homologous and conserved histone acetyltransferase complexes (144). Besides, yeast Chd1 forms complexes with RNA polymerase II and elongation factors Spt5 and Pob3 for the gene transcription (49, 145). Drosophila Chd1 was found to interact with SSRP1, a nuclear protein involved in the transcription regulation (36). Despite no direct binding to AR, mouse and human CHD1 proteins form complexes with AR cofactors, such as NCoR (146), HOXB13, ETV1, and FOXA1 (23), which mediate AR transcriptome changes upon *CHD1* loss. In addition, both mouse and human CHD1 proteins interact with NF-κB, resulting in the activation of inflammatory pathways (25, 27). Given the context-dependent role of CHD1, it is crucial to identify the interactome of CHD1 in different genetic and molecular subsets of prostate cancer. Combined with high-throughput transcriptome and epigenetic profiling, these studies will uncover the molecular basis of CHD1 during cancer development, progression, and response to therapies.

Last but not least, better cancer model systems are needed for studying CHD1’s biology and its impact on drug responsiveness. Unlike other common cancer types, only a small number of human prostate cancer cell lines are available for preclinical studies. They are insufficient to recapitulate the diversity of molecular subtypes and genetic features in human disease. Although *CHD1* loss are frequently found in primary or castration-resistant prostate tumors, none of those prostate cancer cell lines contains homogeneous deletions of *CHD1*. In the past decade, hundreds of patient-derived organoids and xenograft (PDX) models have been generated by multiple institutes and widely used in the prostate cancer research (147, 148). With high fidelity of histopathologic, genomic, and molecular characteristics, they capture the diverse molecular landscape of naïve prostate cancer or CRPC and enable the development and evaluation of biomarker-driven therapy. However, *CHD1* loss or *SPOP* mutations rarely exist in prostate cancer PDX models. De Sarkar et al. recently identified two PDX models, LuCaP78 and LuCaP78CR, lack transcript and protein of CHD1 (90). Both lines, originating from the same patient, contain a combination of monoallelic genomic loss and epigenetic silencing of the remaining allele, show homology-directed DNA repair deficiency features, and are sensitive to IR and carboplatin treatment (90). It remains unclear why prostate tumors with *CHD1* loss and/or *SPOP* mutation have a lower engraftment rate when generating PDX lines, but it is important to establish additional PDX models to mimic this distinct molecular subtype for biology studies and the development of effective therapeutics.

Several GEM models containing prostate-specific *Chd1* deletion have been generated, and provide important tools for investigating CHD1 biology. However, none of them fully recapitulate the genetic and molecular features of prostate cancers with *CHD1* deletion. Conditional knockout of Pten is the most used allele when generating GEM models of prostate cancer, and that’s why most *CHD1* loss GEM models contain PTEN co-deletion (23, 27). These models provide good tools to study the roles of CHD1 in PTEN-deficient tumors, but they showed benign or less aggressive phenotypes due to CHD1’s essentiality in this context. Given the mutual exclusivity between *CHD1* deletion and *PTEN* loss in prostate cancer patients, the co-deletion GEM models couldn’t represent genetic features in human diseases. Efforts have been made to generate GEM models to mimic the molecular subtype of *CHD1* deletion and *SPOP* mutations, but the Chd1/Spop double-knockout mice displayed prostatic intraepithelial neoplasia at 12 months of age and failed to generate prostate adenocarcinoma (89). Using mouse prostate epithelial progenitor/stem cells (PrP/SC) graft model, Cramer’s group showed that co-suppression of CHD1 and MAP3K7 led to high-grade PIN and invasive carcinoma phenotypes (20). It is worth testing whether this combination drives tumorigenesis and progression in Pb-Cre-driven GEM models. Nevertheless, combining the next-generation CHD1 deletion GEM models with cutting-edge single-cell transcriptome profiling will help us fully understand the impact of CHD1 on disease progression, lineage plasticity, response to therapy, and the crosstalk between cancer cells and diverse immune components in the TME. Importantly, the knowledge obtained in prostate cancer will also inform the studies of CHD1 and other CHD remodelers in other cancer types.

## Author contributions

HL and DZ designed the framework of the review. HL, LG, and DZ wrote the manuscript. LG and DZ drew the figures. All authors contributed to the article and approved the submitted version.
